# Supplementary material for: Neck pain associated with smartphone usage among university students
Source: PLoS One. 2023 Jun 23;18(6):e0285451. doi: 10.1371/journal.pone.0285451 (PMC10289365; doi:10.1371/journal.pone.0285451)
Supplement: Attachment — Submitted filename: Cover letter to PLOS ONE Journal Edirors.docx [file pone.0285451.s001.docx]

**PLOS ONE**

March 9, 2023

Re: manuscript re-submission

**Dear** Editor,

We are resubmitting our manuscript**, "Neck pain associated with smartphone use among university students,"** to be considered for publication as an original research article in the Healthcare Journal.

We thank you and the reviewers for taking the time to read our paper and providing valuable comments. It was your valuable and insightful comments that led to possible improvements in the current version. The authors have carefully considered the comments and tried our best to address every one of them. We are hopeful that the manuscript will careful revisions to meet your high standards. The authors welcome further constructive comments, if any.

Below, we provide the point-by-point responses. All modifications to the manuscript have been highlighted in red. Please refer to our submission page and line numbers to see what modifications we've made. The authors provided two copies of the manuscript: one with tracking changes and the other without.

Thank you for your time and consideration of our work. If you have any questions, please contact us.

**Sincerely,**

**Journal Requirements:**

When submitting your revision, we need you to address these additional requirements.

1. Please ensure that your manuscript meets PLOS ONE's style requirements, including those for file naming. The PLOS ONE style templates can be found at

https://journals.plos.org/plosone/s/file?id=wjVg/PLOSOne_formatting_sample_main_body.pdf and 
https://journals.plos.org/plosone/s/file?id=ba62/PLOSOne_formatting_sample_title_authors_affiliations.pdf

2. Please provide additional details regarding participant consent. In the ethics statement in the Methods and online submission information, please ensure that you have specified what type you obtained (for instance, written or verbal, and if verbal, how it was documented and witnessed). If your study included minors, state whether you obtained consent from parents or guardians. If the need for consent was waived by the ethics committee, please include this information.

The followings were added to the ethical approval section. “A written informed consent was obtained on the first page of the study’s questionnaire, which was written in Arabic and English (the official language in Saudi Arabia is Arabic). For participants aged less than18 years, their parents/guardians also provided informed written consent.”

3. We note that you have indicated that data from this study are available upon request. PLOS only allows data to be available upon request if there are legal or ethical restrictions on sharing data publicly. For more information on unacceptable data access restrictions, please see http://journals.plos.org/plosone/s/data-availability#loc-unacceptable-data-access-restrictions.

We are very sorry

The data underlying the results presented in the study have been submitted.

In your revised cover letter, please address the following prompts:

a) If there are ethical or legal restrictions on sharing a de-identified data set, please explain them in detail (e.g., data contain potentially sensitive information, data are owned by a third-party organization, etc.) and who has imposed them (e.g., an ethics committee). Please also provide contact information for a data access committee, ethics committee, or other institutional body to which data requests may be sent.

b) If there are no restrictions, please upload the minimal anonymized data set necessary to replicate your study findings as either Supporting Information files or to a stable, public repository and provide us with the relevant URLs, DOIs, or accession numbers. For a list of acceptable repositories, please see http://journals.plos.org/plosone/s/data-availability#loc-recommended-repositories.

The data underlying the results presented in the study have been submitted.,

4. We note that you have stated that you will provide repository information for your data at acceptance. Should your manuscript be accepted for publication, we will hold it until you provide the relevant accession numbers or DOIs necessary to access your data. If you wish to make changes to your Data Availability statement, please describe these changes in your cover letter and we will update your Data Availability statement to reflect the information you provide.

5. We note you have included a table to which you do not refer in the text of your manuscript. Please ensure that you refer to Table 5 and 6 in your text; if accepted, production will need this reference to link the reader to the Table.

Tables 5 and 6 were references in the result section. Has been corrected to Table 5 instead of Table 3 in the clean copy as per your request, as follows:

“In the univariate model, all hand sides used for writing text messages, time spent using a mobile device, and having a history of neck or shoulder pain were significant predictors of neck and shoulder pain severity (p 0.018) (Table 5). However, in the multivariate model, both having a history of neck or shoulder pain (OR: -0.213, 95% CI: 0.639 -0.748, p<0.001) and the hand-side used for writing (OR: -0.072, 95%CI: -1.18 - -0.081, p=0.025) were significant predictors of neck and shoulder pain severity, and they both explained 11.3% of its variance. (Table 6).”

**The reviewers’ comments were addressed, and the authors responses are below:**

Reviewers' comments:

Reviewer's Responses to Questions

**Comments to the Author**

1. Is the manuscript technically sound, and do the data support the conclusions?

The manuscript must describe a technically sound piece of scientific research with data that supports the conclusions. Experiments must have been conducted rigorously, with appropriate controls, replication, and sample sizes. The conclusions must be drawn appropriately based on the data presented.

Reviewer #1: Partly

Reviewer #2: Yes

We thank the reviewer for this comment. The entire manuscript has been edited to improve the quality and to make the study replicable. More information has been added to explain the study sample size and the discussion and conclusion were modified to reflect the results of the study.

2. Has the statistical analysis been performed appropriately and rigorously?

Reviewer #1: I Don't Know

Reviewer #2: Yes

For the statistical analysis of this study, we consulted a statistician, and he indicated that the statistical analysis used in this study is appropriate and rigorous. In this study, univariate regression model was used to identify factors that predict the outcomes of interest and to evaluate the proportion that each factor can explain the outcomes of interest. While the multivariate regression model was used to determine the best fit model.

3. Have the authors made all data underlying the findings in their manuscript fully available?

The [PLOS Data policy](http://www.plosone.org/static/policies.action#sharing) requires authors to make all data underlying the findings described in their manuscript fully available without restriction, with rare exception (please refer to the Data Availability Statement in the manuscript PDF file). The data should be provided as part of the manuscript or its supporting information, or deposited to a public repository. For example, in addition to summary statistics, the data points behind means, medians and variance measures should be available. If there are restrictions on publicly sharing data—e.g. participant privacy or use of data from a third party—those must be specified.

Reviewer #1: No

Reviewer #2: Yes

The data will be available without restriction

4. Is the manuscript presented in an intelligible fashion and written in standard English?

PLOS ONE does not copyedit accepted manuscripts, so the language in submitted articles must be clear, correct, and unambiguous. Any typographical or grammatical errors should be corrected at revision, so please note any specific errors here.

Reviewer #1: Yes

Reviewer #2: Yes

5. Review Comments to the Author

Please use the space provided to explain your answers to the questions above. You may also include additional comments for the author, including concerns about dual publication, research ethics, or publication ethics. (Please upload your review as an attachment if it exceeds 20,000 characters).

**Responses to Reviewer #1:**

| **Reviewer 1** | **Authors responses** |
| --- | --- |
| For lines 91 and 92, please check out the numbers and the percentages. | Thank you for the most important point.  It has been corrected on page 10, lines 8+9, as follows:  “The overall average of hours per day spent on smartphones revealed a maximum percentage of 465 (53.63%) equal to or greater than 4 hours and the lowest percentage of 402 (46.37%) less than 4 hours.” |
| For lines 96-98, please check out the numbers and the percentages. | **Thank for your kind response**  It has been corrected on page 10, lines 14+15, as follows:  For test massages sent per day, the highest percentage was for those who sent 22 test massages or more 317 (29.99%), followed by 1-5 messages with 260 (29.99%), and the lowest percentages of 12-15 with 32 (3.69%). |
| Please add the word hour(s) to Table 1 for the variable - average time spent with mobile phones. | Thank you for your good comment.  It has been added in to the table 1 |
| Please add the percentages for the variable purpose of use - playing games. | I appreciated your comment.  It has been added on Table 1. |
| Page 12, for lines 238 and 239, please check out the percentages. | Thank for your important comments  It has been corrected on page 15, lines 2- 3, as follows:  In Figure 7, the findings of the current study are shown to be that 292 (33.7%) of the participants had mild pain, 233 (26.9%) had moderate pain, and 89 (10.3%) had severe pain. |
| Page 13, for lines 249-250, it was written “(OR: -250 0.213, 95% CI: -2.357 to -1.268, p<0.001)” for Table 4. Really? | It has been corrected on page 16, lines 2- 6, as follows:  In the multivariate model, only having a history of neck or shoulder pain (OR: 0.647, 95%CI: 0.567 - 0.738, p≤0.001) and the number of hours spent on the device for studying (OR.19, 95% CI: 0.254-0.512, p<0.001) were significant predictors of neck and shoulder pain duration in multivariate model, and they both explained 8.4% of its variance (Table 4). |
| Page 13, for lines 258-260, repetition of the same paragraph which was written above. It is stated that “(OR: -250 0.213, 95% CI: -2.357 to -1.268, p<0.001)” is written in Table 4 for lines 249-250 on page 13. Really? | Thank you very much for your comments.  It has been corrected on page 16, lines 13- 16, as follows:  “in the multivariate model, both having a history of neck or shoulder pain (OR: -0.213, 95% CI: 0.639 -0.748, p<0.001) and the hand-side used for writing (OR: -0.072, 95%CI: -1.18 - -0.081, p=0.025) were significant predictors of neck and shoulder pain severity, and they both explained 11.3% of its variance. (Table 6).” |
| According to me, in the discussion section, links/comparisons to other literature are poor. The discussion section should be rewritten. | Thank you so much for your comments.  The discussion and conclusion sections have been edited and corrected in red to reflect the findings of the current study. |

**Responses to Reviewer #2:**

| **Reviewer 2** | **Authors responses** |
| --- | --- |
| Abstract Section Mention the objectives correctly. | Thank you for the most important point.  The conclusion of the abstract has been corrected on page 2, lines 4-12, as follows:  Objective: Neck and shoulder pain has been linked to prolonged periods of flexed neck posture. However, the influences of factors related to individuals’ characteristics and the time duration and position of using smartphones on the severity and duration of neck and shoulder pain among university students are not well studied. The aim of this study was to identify factors related to individual demographic, history of neck pain, and time duration and positions of using the smart phone that could associate with neck pain severity and duration, and to determine the influence of these factors on neck pain severity and duration among university students. |
| Conclusion in the abstract section should not include the future scope. Mention clinical implication of the current study in the conclusion | Thank for your kind response  The conclusion of the abstract has been corrected on page 3, lines 19-23, as follows:  The results of this study may be utilized to pinpoint smartphone usage factors associated with neck and shoulder pain severity and duration. Further, the findings of this study might help to develop preventive strategies to lower the impacts of these factors on the development of neck and shoulder pain severity and duration among university students |
| **Main Manuscript**  **Methods**  1. Give statistics of how many questionnaires were rolled out and how many filled with Response rate calculation | **Thank you for your good comment**.  It has been edited and added on page 6, lines 23- 24, as follows:  The questionnaires were distributed to the five health faculties by the authors. Questionnaires were distributed to students via Facebook, an online social media and social networking service, by posting them in their batch groups. We received a total of 1000 questionnaires, of which 867 were included and 133 were excluded due to missing data. |
| **2. Exclusion criteria to be mentioned** | I appreciated your comment.  The exclusion criteria have been clarified on page 7, line 23, and page 8, lines 1-2, as follows:  Students were excluded from the study if they had any of the following chronic medical conditions: musculoskeletal disorders or a prior surgery in the neck, shoulder, or upper limb. |
| 3. Process of development of questionnaire and how its validity was done to be mentioned. | Thank for your important comments  It has been added on page 7, lines 14- 16, as follows:  The English version of the survey was reliable and valid. The English version of the survey was translated into Arabic using back translation for the Arabic sample (Kristine Bandgaard 2019). |
| 4. In inclusion criteria, give operational definition for duration of usage of smartphone. | Thank you for your very important comments.  It has been defined on page 7, line 20+ page 8, lines 1- 2 as your requested. |
| Discussion to be written in detail with reason for all the factors to have an impact on neck pain. | Thank you again and again for the very important comments. The discussion was rewriting as you requested |

6. PLOS authors have the option to publish the peer review history of their article ([what does this mean?](https://journals.plos.org/plosone/s/editorial-and-peer-review-process#loc-peer-review-history)). If published, this will include your full peer review and any attached files.

If you choose “no”, your identity will remain anonymous but your review may still be made public.

**Do you want your identity to be public for this peer review?** For information about this choice, including consent withdrawal, please see our [Privacy Policy](https://www.plos.org/privacy-policy).

Reviewer #1: No

Reviewer #2: **Yes:** Prachita Walankar
