# Supplementary material for: Neck pain associated with smartphone usage among university students
Source: PLoS One. 2023 Jun 23;18(6):e0285451. doi: 10.1371/journal.pone.0285451 (PMC10289365; doi:10.1371/journal.pone.0285451)
Supplement: Attachment — Submitted filename: Responses to reviewers_Pols.docx [file pone.0285451.s002.docx]

Reviewers' comments:

Reviewer's Responses to Questions

**Comments to the Author**

1. If the authors have adequately addressed your comments raised in a previous round of review and you feel that this manuscript is now acceptable for publication, you may indicate that here to bypass the “Comments to the Author” section, enter your conflict of interest statement in the “Confidential to Editor” section, and submit your "Accept" recommendation.

Reviewer #1: (No Response)

Reviewer #2: All comments have been addressed

Reviewer #3: (No Response)

2. Is the manuscript technically sound, and do the data support the conclusions?

The manuscript must describe a technically sound piece of scientific research with data that supports the conclusions. Experiments must have been conducted rigorously, with appropriate controls, replication, and sample sizes. The conclusions must be drawn appropriately based on the data presented.

Reviewer #1: Yes

Reviewer #2: Yes

Reviewer #3: (No Response)

3. Has the statistical analysis been performed appropriately and rigorously?

Reviewer #1: I Don't Know

Reviewer #2: Yes

Reviewer #3: No

4. Have the authors made all data underlying the findings in their manuscript fully available?

The [PLOS Data policy](http://www.plosone.org/static/policies.action#sharing) requires authors to make all data underlying the findings described in their manuscript fully available without restriction, with rare exception (please refer to the Data Availability Statement in the manuscript PDF file). The data should be provided as part of the manuscript or its supporting information, or deposited to a public repository. For example, in addition to summary statistics, the data points behind means, medians and variance measures should be available. If there are restrictions on publicly sharing data—e.g. participant privacy or use of data from a third party—those must be specified.

Reviewer #1: Yes

Reviewer #2: Yes

Reviewer #3: (No Response)

5. Is the manuscript presented in an intelligible fashion and written in standard English?

PLOS ONE does not copyedit accepted manuscripts, so the language in submitted articles must be clear, correct, and unambiguous. Any typographical or grammatical errors should be corrected at revision, so please note any specific errors here.

Reviewer #1: Yes

Reviewer #2: Yes

Reviewer #3: Yes

6. Review Comments to the Author

Please use the space provided to explain your answers to the questions above. You may also include additional comments for the author, including concerns about dual publication, research ethics, or publication ethics. (Please upload your review as an attachment if it exceeds 20,000 characters)

Reviewer #1: Why the sentences are different which explain the aim of the study in the abstract and the introduction section?

The aim of the study has been corrected and it is being consistent in both the abstract and intro section as the following:

Page 6, lines 7-11, as follows:

The aim of this study was to identify factors related to individual demographics, the history of neck pain, and the time duration and positions of using the smartphone that could be associated with neck pain severity and duration and to determine the influence of these factors on neck pain severity and duration among university students.

Page 12, Line 22, I suggest, 317 (36.56%)

Page 10, line 8.

It has been corrected, as per your request.

The conclusion section should be clear and concise.

Conclusions: The findings of this study may help academic institutions and collegiate students be aware of the negative impact of using smartphones and identify those measures that can contribute to the development of neck and shoulder pain duration and severity. Furthermore, academic institutions may consider providing students with study materials that require using a smartphone for a short time in an attempt to lower the risk and severity of musculoskeletal pain among the young academic population.

These two sentences have been deleted.

Reviewer #2: Yes. The authors have answered to all queries raised during first review. This has enhanced the quality of study.

Reviewer #3: The goal of this manuscript intended to identify factors associated with neck pain severity and duration among university students using smartphone. They conduct a cross-sectional study with online questionnaire and analyzed 867 responses. They reported the significant association of neck and shoulder pain duration or severity with a history of neck or shoulder pain, the hand-side used for writing, and the number of hours spent on the device for studying.

1. Line 11 in page 9. “linear regression model, OR: odds ratios…” how to present OR in linear regression?

We apologize for the mistake, the odd ratios have been removed from the tables and the manuscript.

2. Table 1. What’s the total time for “The average time spent using a mobile phone (hour)”? Per day, per week, or others? Same question applies to other parts of the table.

The questions asked about the number of hours spent on smartphones and the number of texts sent per day. The table shows the number of students stratified by the number of hours, and we did not collect data about the total number of hours.

3. Table 1. are “playing games”, “social media”, and “studying” exclusive? i.e. it should be “playing games only” and so on?

Thank you for your comment, but table 1 represents the questionnaire as is.

4. Tables 3, 4, 5, and 6. What analysis was performed for the results in Tables 3, 4, 5, and 6? The title says “linear regression”. If it is linear regression, what’s OR presented in the table? The same question applies to the corresponding manuscript text as well!

The statistical analysis used was linear regression, and we agree with the reviewer that the Odd ratio is not appropriate to be used in this case and the β coefficients are the regression coefficients that are appropriate to be used to indicate the difference between two marginal means in continuous measures. The odd ratios have been removed from the tables and the manuscript.

7. PLOS authors have the option to publish the peer review history of their article ([what does this mean?](https://journals.plos.org/plosone/s/editorial-and-peer-review-process#loc-peer-review-history)). If published, this will include your full peer review and any attached files.


If you choose “no”, your identity will remain anonymous but your review may still be made public.


**Do you want your identity to be public for this peer review?** For information about this choice, including consent withdrawal, please see our [Privacy Policy](https://www.plos.org/privacy-policy).

Reviewer #1: No

Reviewer #2: No

Reviewer #3: No
